# Molecular characterization of methicillin-resistant *Staphylococcus aureus* among insulin-dependent diabetic individuals in Brazil

**DOI:** 10.1186/s12941-020-00401-y

**Published:** 2021-02-10

**Authors:** Nathalia Bibiana Teixeira, Carlos Magno Castelo Branco Fortaleza, Matheus Cristovam de Souza, Thais Aline Monteiro Pereira, Bibiana Prada de Camargo Colenci, Maria de Lourdes Ribeiro de Souza da Cunha

**Affiliations:** 1 Departamento de Infectologia, Dermatologia, Diagnóstico Por Imagem e Radioterapia, Faculdade de Medicina de Botucatu, UNESP - Universidade Estadual Paulista Júlio de Mesquita Filho, Botucatu, SP Brasil; 2 Departamento de Ciências Químicas e Biológicas, Instituto de Biociências de Botucatu, UNESP - Universidade Estadual Paulista Júlio de Mesquita Filho, Botucatu, SP Brasil; 3 Departamento de Clínica Médica – Endocrinologia, UNESP - Universidade Estadual Paulista Júlio de Mesquita Filho, Botucatu, SP Brasil; 4 Departamento de Ciências Químicas e Biológicas – Setor Microbiologia e Imunologia, Instituto de Biociências de Botucatu (IBB)-Laboratório de Bacteriologia. Rua Plínio Silva, CEP: 18618-970 – Distrito de Rubião Júnior, Botucatu, SP Brasil

**Keywords:** Methicillin-resistant *Staphylococcus aureus* (MRSA), Diabetes mellitus, Insulin, Resistance, Nasal or oropharyngeal carriage, Molecular typing

## Abstract

**Background:**

People with diabetes mellitus, especially insulin-dependent diabetic patients, are a risk group for staphylococcal infections. Asymptomatic infection with *Staphylococcus aureus* is common and favors dissemination of the microorganism, rendering these individuals a source of infection. This study aimed to characterize the resistance profile, clonal profile and sequence type, as well as to analyze the prevalence and risk factors for nasal and oropharyngeal carriage of methicillin-susceptible (MSSA) and methicillin-resistant *S. aureus* (MRSA) isolated from insulin-dependent diabetic individuals in the city of Botucatu, SP, Brazil.

**Methods:**

*Staphylococcus aureus* was collected from the nasopharynx and oropharynx of 312 community-dwelling insulin-dependent diabetic individuals over a period of 3 years (October 2015 to December 2018). The isolates were characterized by susceptibility profiling, detection of the *mec*A gene, SCC*mec* typing, and molecular typing by PFGE and MLST. The risk factors associated with *S. aureus* and MRSA carriage were determined by logistic regression analysis.

**Results:**

The overall prevalence of colonization with *S. aureus* and MRSA was 30.4% and 4.8%, respectively. Fifteen of the 112 *S. aureus* isolates carried the *mec*A gene; SCC*mec* type IV was identified in 10 isolates, SCC*mec* type I in three, and SCC*mec* type II in two. Among the 15 resistant isolates (MRSA), four were susceptible to oxacillin/cefoxitin by the disc diffusion method and one MSSA isolate was resistant to sulfamethoxazole/trimethoprim. The analysis of risk factors revealed a protective effect of age and lung disease, while lower-extremity ulcers were a risk factor for *S. aureus*. For MRSA, only male gender was significantly associated as a risk factor in multivariate analysis. Clonal profile analysis demonstrated the formation of *clusters* among MRSA isolates from different patients, with the identification of ST5-IV, ST5-I, and ST8-IV. Isolates carrying ST398 were identified among MSSA and MRSA (ST398-IV).

**Conclusion:**

Our findings reinforce the importance of epidemiological studies of *S. aureus* carriage, especially in populations at high risk of infections such as diabetics. The data suggest widespread dissemination of MRSA in the population of insulin-dependent diabetic patients studied, as well as the emergence of important lineages among these individuals.

## Background

Diabetes mellitus is a progressive chronic disease characterized by high blood glucose levels, which is one of the most prevalent diseases in modern societies. Its treatment is often inadequate or absent [1]. It is estimated that more than 370 million people worldwide have diabetes and more than 5 million deaths were attributed to the disease and its complications in 2017 [2, 3].

People with diabetes are known to be more susceptible to infections because of their elevated blood glucose levels and suppression of the immune response. In addition, neuropathy and reduced blood flow to the extremities are common in these individuals. Consequently, wounds tend to heal more slowly, increasing the risk of amputations and death [4, 5].

*Staphylococcus aureus* is one of the leading causes of infections in diabetic individuals. This microorganism plays a particularly important role in this scenario by causing infections that range from superficial to severe and potentially fatal systemic infections, in addition to its ability to acquire resistance to multiple drugs. In the past, the pathogen was mainly found in hospital settings; however, we are now witnessing an increase particularly of resistant isolates (MRSA) acquired in the community that are genetically different from traditional nosocomial strains [6–8]. Infections caused by methicillin-resistant *S. aureus* (MRSA) are associated with a higher mortality rate compared to infections caused by methicillin-susceptible *S. aureus* (MSSA) [2]. Furthermore, studies suggest that staphylococcal infections are preceded by colonization with these microorganisms [2, 9–13] and at least one third of colonized healthy adults are at risk of developing subsequent invasive infections [6, 7].

Bacterial resistance has become a major global threat. In the study of Onanuga and Temedie [14], the prevalence of multidrug-resistant MRSA isolated from the anterior nares of diabetic patients was 52.5%. This prevalence was 40% among diabetic outpatients in the study of Kutlu et al. [15]. High antimicrobial resistance of MRSA isolated from diabetic patients has also been reported by Alizargar et al. [16], with rates of erythromycin, ciprofloxacin and clarithromycin resistance of 81.9%, 71.3% and 65.5%, respectively, and a multidrug resistance rate of 59%. Two vancomycin-resistant *Staphylococcus aureus* strains were isolated in that study [16].

Diabetic individuals are considered a risk group for skin infections such as those caused by *S. aureus* and are also more prone to developing severe systemic infections [13]. Studies have shown that the prevalence of nasal colonization with *S. aureus* (27–56.6%) and MRSA (1–7.3%) varies according to geographic location and that the use of insulin is a risk factor for nasal MRSA carriage in diabetic individuals [16]. However, little is known about the role of throat colonization in this population.

*Staphylococcus aureus* is characterized by a high adaptation potential demonstrated by its success in surviving in different environments and the constant emergence of new lineages that cause different clinical manifestations and exhibit rapid epidemiological dissemination, causing a significant impact on health [17]. Globally disseminated MRSA clones included healthcare-associated [HA]-MRSA. The prevalence of this microorganism has also been increasing among community-associated [CA]-MRSA and livestock-associated [LA]-MRSA infections [17]. However, studies have demonstrated an exchange of these lineages with CA-MRSA, causing outbreaks of healthcare-associated infections in the community, and HA-MRSA being isolated in community settings [18, 19]. LA-MRSA have also been reported in individuals without any previous contact with animals [17].

Staphylococcal cassette chromosome (SCC*mec*) typing is a useful epidemiological tool since different types are more prevalent in hospital or community settings. Unlike HA-MRSA that harbor large SCC*mec* (SCC*mec* types I, II and III) and are frequently multidrug-resistant, CA-MRSA are associated with SCC*mec* types IV and V, which are smaller and only carry the *mec*A gene of methicillin resistance [20–23].

In view of their complex epidemiology, *S. aureus* and MRSA lineages have been identified by techniques such as pulsed-field gel electrophoresis (PFGE), an adequate tool for the study of local outbreaks. Studies investigating the clonality of *S. aureus* are frequently complemented by multilocus sequence typing (MLST), which permits comparisons between *S. aureus* sequences described in different parts of the world. MLST and SCC*mec* typing have been used for the identification of global MRSA clones, which resulted in the detection of five large MRSA clonal complexes (CC) including CC5, CC8, CC30, CC45, and CC398 [24, 25].

Considering their impact on the health of diabetic individuals, a better understanding of the epidemiology of and risk factors for colonization with *S. aureus* and MRSA is necessary. The aim of this study was to characterize the resistance profile, clonal profile and sequence type, as well as to analyze the prevalence and risk factors for nasal and oropharyngeal carriage of MSSA and MRSA isolated from insulin-dependent diabetic individuals in the city of Botucatu, SP, Brazil.

## Materials and methods

### Study design

This was a cross-sectional study conducted in the city of Botucatu, São Paulo, Brazil, whose estimated population is 146,497 inhabitants [26]. We calculated the sample size from a population of 1631 individuals using an expected proportion of 50% (the typical value used in situations in which the proportion is unknown), considering a power of 80% (i.e., a beta error of 20%) and an alpha error of 5%, with a design effect of 1.0 (since no subsampling was performed). The formula used for sample size calculation is given in Additional file 1: Appendix S1. Using this calculation for proportions, we obtained a suggested *n* of 312 subjects. The subjects were selected randomly from the database of the Municipal Health Department over a period of 3 years (October 2015 to December 2018). If possible, the subjects were recruited during home visits (n = 204); however, in view of the difficulty in locating these individuals at the addresses obtained, many of them were invited by telephone to the events promoted at Basic Health Units (n = 70). Some subjects were recruited at the doctor’s offices (n = 23) and at the headquarter of the Botucatu Association for Diabetes Support (ABAD in the Portuguese acronym) (n = 15). A questionnaire including the following data was applied to subjects who agreed to participate in the study: demographic data (gender and age); type of diabetes (1 or 2); time since diagnosis (years); time of insulin use (years); clinical data (comorbidities); presence of ulcers or amputations; tattoos; hospitalizations or medical procedures in the last year; and use of antimicrobials in the last year. All data were obtained by interview with the patient and/or legal representative following ethical standards. The questionnaires were extensively reviewed for inconsistencies.

### Inclusion and exclusion criteria

All insulin-dependent diabetic patients living in the city of Botucatu, who agreed to participate by signing the free informed consent form (Additional file 1: Table S1), were included in the study. Patients who did not consent to participate in the study, patients who did not use insulin at the time of data collection, patients who had died, and patients who could not be found based on their personal data were excluded (Additional file 1: Table S2).

### Collection of microbiological specimens

Nasal and oral mucosa samples were collected from 312 insulin-dependent diabetic individuals living in the city of Botucatu, São Paulo, Brazil, using sterile swabs in transport medium. Samples from the anterior nares and oropharynx were obtained using one swab for each site. For the collection of nasal samples, the swab was immersed in 0.9% sterile saline and inserted into both nares, rotating it and gently pressing its end against the mucosa. The technique for oropharyngeal sampling consisted of immersion of the swab and passing it gently over the surface of the throat, avoiding contact of the examiner with the tongue.

The swabs were transported in Stuart medium to the Laboratory of Bacteriology, Department of Microbiology and Immunology, Institute of Biosciences, UNESP, and seeded onto plates containing Baird-Parker agar, a selective medium for Staphylococcus. After incubation for 48 h at 37 °C, the isolated microorganisms were identified.

### Identification of *Staphylococcus aureus*

The microorganisms were submitted to Gram staining for observation of their morphology and specific staining. After confirmation of these features, catalase and coagulase tube tests and biochemical tests (maltose, trehalose, and mannitol) were carried out to differentiate *S. aureus* from other *Staphylococcus* species [27, 28].

After DNA extraction with the Illustra Kit (GE Healthcare, Little Chalfont, Buckinghamshire, UK), the *S. aureus* isolates were confirmed genotypically by detection of the 16S rRNA gene [29] and the DNA SA442 fragment specific for *S. aureus* [30]. Thus, 112 *S. aureus* isolated obtained from 312 patients included in the study were identified.

### Antimicrobial susceptibility testing

All 112 isolates obtained were subjected to antimicrobial susceptibility testing by the disc diffusion method using impregnated discs according to the criteria of the Clinical Laboratory Standards Institute (CLSI) [31]. The inoculum was adjusted to a 0.5 McFarland standard and seeded onto Mueller–Hinton agar and the plates were incubated for 24 h at 35 ºC. The following drugs were used: oxacillin (1 µg), cefoxitin (30 µg), linezolid (30 µg), quinupristin/dalfopristin (15 μg), and sulfamethoxazole/trimethoprim (25 µg). Antimicrobial activity was evaluated by determining the diameter of the inhibition zone, which was interpreted according to the CLSI [31]. International reference strains were used as controls for MSSA (*S. aureus* ATCC 25923) and MRSA (*S. aureus* ATCC 33591).

### Determination of the minimum inhibitory concentration

The minimum inhibitory concentration (MIC) of vancomycin was determined by the E-test in all 112 *S. aureus* isolates. This quantitative test uses inert and transparent plastic strips (60 mm long × 5.5 mm wide) with a predefined gradient of concentrations of the antimicrobial to be tested. The MIC results were classified as susceptible, intermediate, or resistant according to the definitions of the CLSI [31]. An international reference strain (*S. aureus* ATCC 29213) was included as control.

### Detection of the *mec*A gene of methicillin resistance

All *S. aureus* isolates were analyzed regarding the presence of the *mec*A gene by PCR following the parameters described by Murakami et al. [32]. International reference strains were included as positive (*S. aureus* ATCC 33591) and negative (*S. aureus* ATCC 25923) controls in all reactions. All isolates in which the *mec*A gene was detected were classified as MRSA, regardless of the result of the oxacillin and cefoxitin disc diffusion tests.

### Determination of staphylococcal cassette chromosome *mec* (SCC*mec*) type

SCC*mec* typing was performed by multiplex PCR as described by Oliveira and de Lencastre [33] and updated by Milheiriço et al. [34] on all *mec*A gene-positive *S. aureus* isolates. The following strains were used as controls: COL for SCC*mec* type I; N315 for SCC*mec* type IA; PER34 for SCC*mec* type II; AN546 for SCC*mec* type III; HU25 for SCC*mec* type IIIA, and MW2 for SCC*mec* type IV.

### Visualization of amplified products

The efficiency of the amplifications was confirmed by electrophoresis on 2% agarose gel prepared in 0.5 M Tris–borate-EDTA (TBE) buffer. A 100-bp marker was used as molecular weight standard. The gel was stained with SYBR® Safe and photographed under UV transillumination.

### Pulsed field gel electrophoresis (PFGE)

The modified protocol of McDougal et al. [35] was used to type all 112 *S. aureus* isolates by PFGE. The isolates were grown in BHI broth for 24 h. Next, 400 µL of the sample was added to a microtube and centrifuged at 12,000 rpm for 50 s. The supernatant was discarded and 300 µL TE solution (10 mM Tris, 1 mM EDTA, pH 8.0) was added. The samples were kept in a water bath for 10 min at 37 °C. After vortexing, 5 µL lysostaphin (1 mg/mL in 20 mM sodium acetate, pH 4.5) and 300 μL low-melting agarose were added.

The samples were transferred to plug molds. The plugs were allowed to solidify and then placed in a 24-well plate with 2 mL EC solution (6 mM Tris–HCl, 1 M NaCl, 100 mM EDTA, 0.5% Brij-58, 0.2% sodium deoxycholate, 0.5% sodium lauroyl sarcosine) and incubated at 37 °C for at least 4 h. The EC solution was removed and the plugs were washed four times with 2 mL TE at room temperature at intervals of 30 min.

The *SmaI* enzyme (Fast Digest *SmaI,* Life Science, Canada) was used for restriction of genomic DNA. Electrophoresis was carried out in a CHEF-DR III System (BioRad Laboratories, USA) on 1% agarose gel prepared with 0.5 M TBE (Pulsed Field Certified Agarose, BioRad Laboratories, USA) under the following conditions: pulse switch time of 5 to 40 s for 21 h using a linear ramp; 6 V/cm; angle of 120°; 14 ^o^ C; 0.5 M TBE as running buffer. The Lambda PFG Ladder (New England BioLabs) was used as molecular marker. The gel was stained with GelRed (10,000× in water; Biotium, USA) for 1 h and photographed under UV transillumination.

Similarity was analyzed with the BioNumerics 7.6 software (Applied Maths, Belgium). The dendrogram was constructed by the UPGMA method (Unweighted Pair Group Method with Arithmetic Mean), with band position tolerance and optimization adjusted to 1.2% and 1%, respectively.

Forty-five isolates, including one MRSA, could not be typed with the *SmaI* enzyme and were therefore digested with the *ApaI* restriction enzyme.

A similarity coefficient ≥ 80% was chosen for the determination of *clusters*. A *cluster* was defined as a group of three or more isolates showing ≥ 80% similarity.

### Multilocus sequence typing (MLST)

Lineages representative of the *clusters* obtained by PFGE were selected for MLST. Five MRSA isolates obtained PFGE-*SmaI* and four MSSA isolates obtained by PFGE-*ApaI* were chosen.

MLST was performed according to the protocol of Enright et al. [36] by amplification and sequencing of seven housekeeping genes (*arcC*, *aro*E, *glp*F, *gmk*, *pta*, *tpi*, and *yqi*L). The PCR products were purified using the HiYield™ Gel/PCR Fragments Extraction Kit and the sequencing reactions were performed in an ABI3500 8-capillary sequencer (50 cm) using POP7 as polymer (Applied Biosystems). The BioNumerics 7.6 program (Applied Maths, Belgium) was used for visualization of the sequences (electropherogram). The sequences were analyzed and compared to an online database (http://www.mlst.net) (2004).

### Statistical analysis

The Epi-Info for Windows software (version 7.26; ©Centers for Disease Control and Prevention, Atlanta, USA) was used for univariate analysis. Dichotomous variables were compared using nonparametric tests of proportion, X^2^ test, and Fisher’s exact test. Numerical variables were compared by the Mann–Whitney U test.

Multivariate analysis was performed with the SPSS 20.0 software (©SPSS, Inc.) using a logistic regression model. The outcomes of interest were the overall presence of *S. aureus* or the presence of MRSA irrespective of sampling site. The variables were selected using a backward stepwise strategy. The criterion for entry and permanence of the variables in the models was p < 0.1. Final statistical significance was set at p < 0.05.

## Results

### Prevalence of *Staphylococcus aureus* and MRSA carriage

**Fig. 1:**
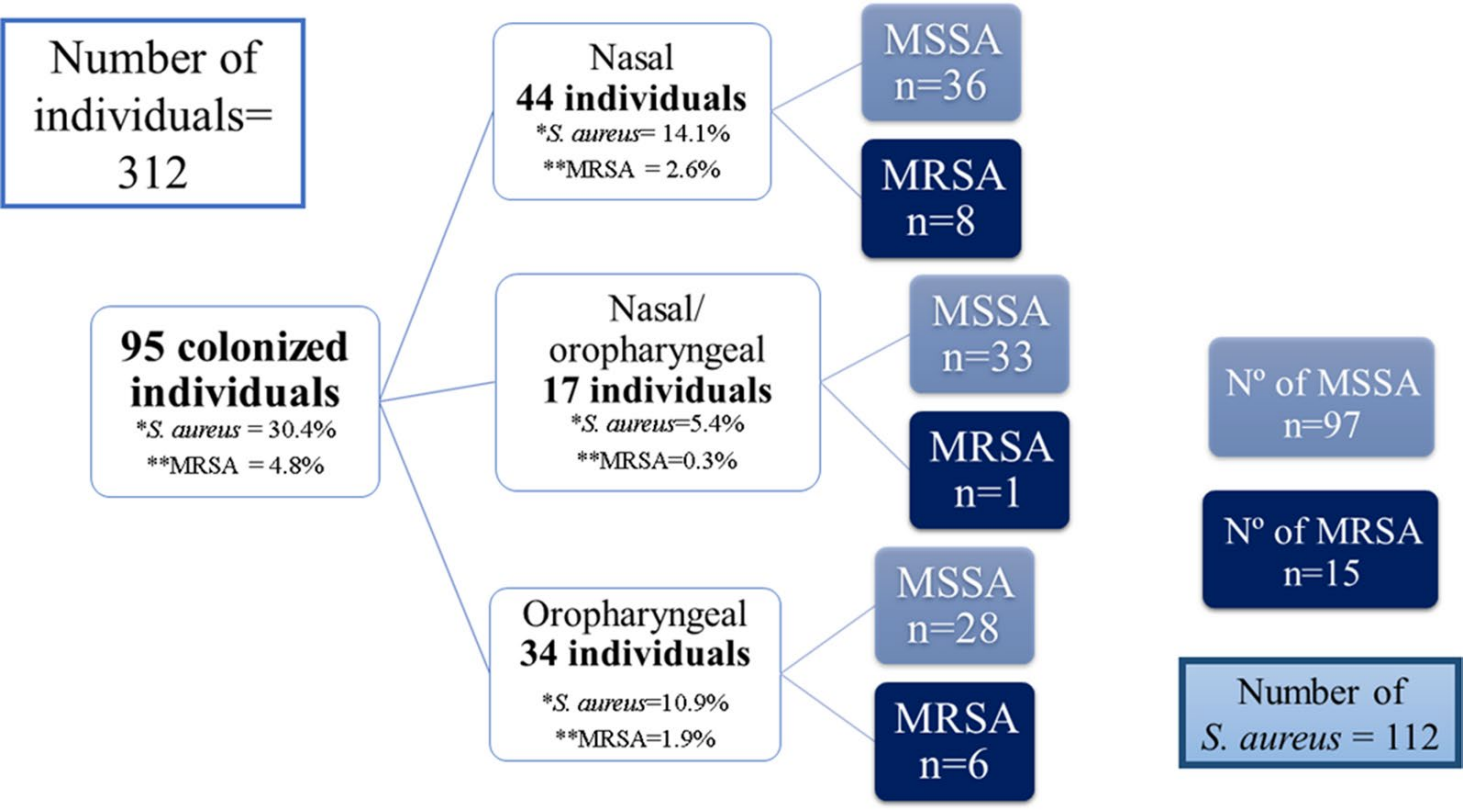
Flow chart of the total number of individuals colonized with *Staphylococcus aureus* and number of individuals colonized with MSSA and MRSA according to sampling site. MSSA, methicillin-susceptible *S. aureus*; MRSA, methicillin-resistant *S. aureus*. *Overall prevalence of *S. aureus*. ** Prevalence of MRSA

Ninety-five of the 312 subjects included in the study were colonized with *S. aureus*, corresponding to an overall prevalence of 30.4% (95%CI 25.6–35.8%). Fifteen of the 95 subjects were colonized with MRSA either in the nose and/or oropharynx, corresponding to a prevalence of 4.8% (95%CI 2.9–7.8%). Regarding the sampling site, *S. aureus* was isolated exclusively from the nose in 44 (14.1%) individuals, exclusively from the oropharynx in 34 (10.9%), and from both sites in 17 (5.4%). Analysis of MRSA demonstrated that eight of the 15 colonized individuals carried the microorganism exclusively in the nose and six exclusively in the oropharynx. Interestingly, one subject carried MSSA in the oral mucosa and MRSA in the nasal mucosa (Fig. 1).

### Determination of in vitro antimicrobial susceptibility

All 112 isolates identified as *S. aureus* were submitted to in vitro antimicrobial susceptibility testing as previously described. The disc diffusion method revealed 11 strains that were resistant to oxacillin and cefoxitin, including seven resistant to both drugs and four that were resistant only to cefoxitin (inhibition zone ≤ 21). It should be noted that four of the isolates that were susceptible to both drugs exhibited resistance by the genotypic method.

Among all isolates, only one was resistant to sulfamethoxazole/trimethoprim and there was no case of resistance to quinupristin/dalfopristin or linezolid. In addition, the MIC50 and MIC90 for vancomycin were 0.50 and 1.0 μg/mL, respectively, and all isolates were susceptible (Supplementary Material Table S3).

### Detection of the *mec*A gene and characterization of SCC*mec*

Fifteen of the 112 *S. aureus* isolates carried the *mec*A gene of methicillin resistance. There was a predominance of the SCC*mec* type IV among isolates (n = 10), but three isolates harboring SCC*mec* type I and two harboring SCC*mec* type II were also identified. Most individuals harboring MRSA isolates reported no contact with the hospital environment in the last year, except for one subject with SCC*mec* type II and one with SCC*mec* type IV.

### Risk factors for *S. aureus* and MRSA carriage

All patients in the study used insulin and 78 (82.1%) had type 2 diabetes. A total of 198 (63.4%) also used other hypoglycemic drugs. The incidence of complications was high: 132 (42.3%) patients had retinopathy and 31 (9.9%) renal impairment. Thirty-one (9.9%) patients reported lower-extremity ulcers and 10 of them [3.2%] had undergone amputation.

The results of univariate and multivariate (logistic regression model) analysis to identify risk factors for *S. aureus* and MRSA carriage are shown in Tables 1 and 2. With respect to risk factors for *S. aureus* carriage, univariate analysis showed a protective effect (median 61 years, *p* = 0.04), heart disease (OR = 0.51, 95%CI 0.28–0.95, *p* = 0.03) and lung disease (OR = 0.28, 95%CI 0.10–0.83, *p* = 0.02), while only the presence of lower-extremity ulcers was a risk factor (OR = 2.36, 95%CI 1.11–5.01, *p* = 0.02). However, the only factors that remained independently associated with *S. aureus* carriage in insulin-dependent diabetic individuals were age (OR = 0.98, 95% CI 0.93–0.99, *p* = 0.02) and lung disease (OR = 0.31, 95% CI 0.10–0.92, *p* = 0.03), which were protective. The only risk factor was leg ulcer (OR = 2.44, 95%CI 1.11–5.34, *p* = 0.03).

**Table 1:** Uni- and multivariate (logistic regression) analysis of predictors of *Staphylococcus aureus* carriage in diabetic individuals

Factor	*S. aureus *(n = 95)	Negative (n = 217)	Univariate analysis		Logistic regression (multivariate)	
			OR (95%CI)	*p*	OR (95%CI)	*p*
Male gender	42 (44.2)	98 (45.2)	0.96 (0.59–1.56)	0.88		
*Age (median, quartiles)*	*61 (49–70)*	*65 (56–71)*	–	*0.04*	*0.98 (0.93–0.99)*	*0.02*
Time since diagnosis, years (median, quartiles)	14 (9–20)	16.5 (8–25)	–	0.84		
Use of insulin, years (median, quartiles)	8 (4–14)	7 (3–11)	–	0.22		
Diabetes type 2	78 (82.1)	184 (84.8)	0.82 (0.43–1.56)	0.35		
*Heart disease*	*16 (17.4)*	*61 (29.0)*	*0.51 (0.28–0.95)*	*0.03*		
*Lung disease*	*4 (4.3)*	*29 (13.8)*	*0.28 (0.10–0.83)*	*0.02*	*0.31 (0.10–0.92)*	*0.03*
Kidney disease	23 (25.0)	59 (28.1)	0.85 (0.49–1. 49)	0.58		
Liver disease	9 (9.8)	18 (8.6)	1.16 (0.50–2.68)	0.73		
CNS disease	19 (20.7)	31 (14.8)	1.50 (0.80–2.82)	0.20		
Cancer	11 (11.8)	28 (13.1)	0.89 (0.42–1.87)	0.75		
Trauma	8 (8.7)	23 (11.0)	0.77 (0.33–1.80)	0.55		
Tattoo	4 (4.3)	12 (5.7)	0.75 (0.23–2.39)	0.78		
*Lower-extremity ulcers*	*15 (16.3)*	*16 (7.6)*	*2.36 (1.11–5. 01)*	*0.02*	*2.44 (1.11–5.34)*	*0.03*
Amputation	2 (2.2)	8 (3.8)	0.56 (0.18–2.70)	0.73		
Charlson comorbidity index ≥ 1	3 (2–4)	3 (2–4)	-	0.84		
Hospitalization in the last year	11 (12.0)	37 (17.6)	0.63 (0.31–1.31)	0.21		
Surgery in the last year	10 (10.9)	22 (10.5)	1.04 (0.47–2.30)	0.91		
Antimicrobial use in the last year	21 (22.8)	57 (27.3)	0.79 (0.44–1.40)	0.42		

All values are reported as number (%) unless otherwise specified. Significant associations are indicated in italic

*OR* odds ratio, *CI* confidence interval, *CNS* central nervous system

**Table 2:** Uni- and multivariate (logistic regression) analysis of predictors of MRSA carriage in diabetic individuals

Factor	MRSA (n = 15)	Negative (n = 287)	Univariate analysis				Logistic regression (multivariate)		
			OR (95%CI)	*p*			OR (95% CI)		*p*
*Male gender*	*11 (73.3)*	*129 (43.3)*	*3.58 (1.11–11.51)*	*0.02*			*3.64 (1.12–11.78)*		*0.03*
Age (median, quartiles)	58 (46–73)	63 (54–71)	–	0.27					
Time since diagnosis, years (median, quartiles)	13 (10–19)	15 (9–24)	–	0.56					
Use of insulin, years (median, quartiles)	8 (3–15)	7 (3–12)	–	0.94					
Diabetes type 2	11 (73.3)	251 (84.5)	0.50 (0.15–1.65)	0.27					
Heart disease	3 (20.0)	74 (25.8)	0.72 (0.20–2.62)	0.77					
Lung disease	0	33 (11.5)	–	0.39					
Kidney disease	4 (26.7)	78 (27.2)	0.97 (0.30–3.15)	1.00					
Liver disease	1 (6.7)	26 (9.1)	0.72 (0.09–5.67)	1.00					
CNS disease	1 (6.7)	49 (17.1)	0.35 (0.04–2.70)	0.48					
Cancer	2 (13.3)	37 (12.7)	1.06 (0.30–4.87)	1.00					
Trauma	0	31 (10.8)	–	0.38					
Tattoo	1 (6.7)	15 (5.2)	1.29 (0.16–10.52)	0.57					
lower-extremity ulcers	3 (20.0)	28 (9.8)	2.31 (0.61–8.69)	0.19					
Amputation	1 (6.7)	9 (3.1)	2.21 (0.26–18.65)		0.40				
Charlson comorbidity index ≥ 1	3 (2–4)	3 (2–4)	–		0.41				
Hospitalization in the last year	2 (13.3)	46 (16.0)	0.81 (0.18–3.69)		1.00				
Surgery in the last year	3 (20.0)	29 (10.1)	2.22 (0.59–8.34)		0.20				
Antimicrobial use in the last year	4 (26.7)	74 (25.9)	1.04 (0.32–3.37)		1.00				

All values are reported as number (%) unless otherwise specified. Significant associations are indicated in italic

*OR* odds ratio, *CI* confidence interval, *CNS* central nervous system

The study of risk factors for MRSA carriage revealed only male gender as a risk factor in uni- and multivariate analysis (OR = 3.64, 95% CI 1.12–11.78, *p* = 0.03).

### Determination of the clonal profile of *S. aureus* and MRSA by pulsed-field gel electrophoresis (PFGE)

A total of 112 *S. aureus* isolates were analyzed by PFGE. Forty-five of these isolates could not be typed repeatedly with *SmaI*, including one MRSA isolate. However, molecular typing of all 45 isolates was possible using the *ApaI* enzyme.

For clonal profile analysis, one dendrogram was constructed for susceptible *S. aureus* isolates (MSSA) and one for resistant isolates (MRSA) using *SmaI* and *ApaI*, which permitted to identify *clusters* with similarity ≥ 80% in both groups.

**Fig. 2:**
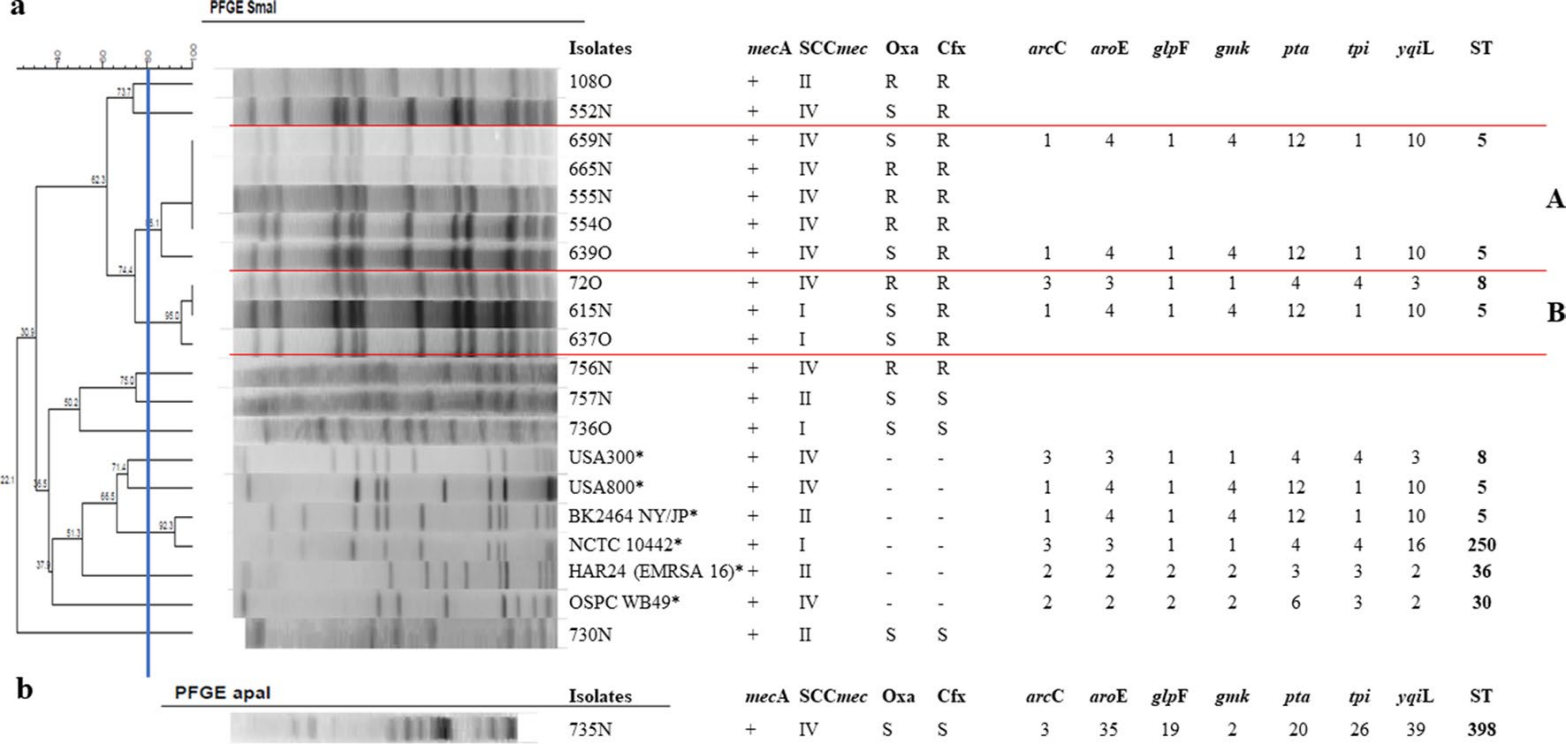
Dendrogram of the PFGE-*SmaI* and PFGE-*ApaI* profiles of MRSA isolated from insulin-dependent diabetic individuals generated by Dice analysis/UPGMA (BioNumerics, Applied Maths) and their molecular characterization by SCC*mec* typing and MLST.** a.** Isolates showing > 80% similarity (*clusters* A and B) after digestion with *SmaI*. **b.** Band pattern of strain 735 N obtained by digestion with *ApaI*. N, nasal mucosa; O, oropharyngeal mucosa; S, susceptible; R, resistant. * International clones used as controls

Figure 2 shows the dendrogram of the PFGE-*SmaI* and PFGE-*ApaI* profiles of MRSA isolates, as well as their in vitro susceptibility profile to oxacillin and cefoxitin, presence of the *mec*A gene, SCC*mec* type and MLST analysis. Analysis of PFGE-*SmaI* isolates revealed the presence of two *clusters* (A and B). *Cluster* A contained five isolates, four of them showing 100% similarity (554O, 555 N, 659 N, and 665 N). All of them were isolated from different individuals and harbored SCC*mec* type IV. In *cluster* B, it is possible to observe two isolates carrying SCC*mec* type I (615 N and 637O) and one isolate carrying SCC*mec* type IV (72O). The MRSA isolate typed with *ApaI* is shown in Fig. 2b. None of the isolates grouped with international clones.

Two *clusters* (A and C) containing four strains and two *clusters* (B and D) containing strains were obtained for the MSSA isolates (Supplementary Material Figure S1). *Cluster* A contained two isolates from the nasal and oropharyngeal mucosa of the same subject, demonstrating colonization of different sites with the same isolate. Similarly, in *cluster* B, two isolates from the nasal and oropharyngeal mucosa of the same subject were grouped with one nasal isolate of another subject. On the other hand, the four isolates of *cluster* C were obtained from unrelated individuals and in *cluster* D all isolates were from the oral mucosa of different subjects. These findings suggest widespread dissemination of *S. aureus* in the community.

Nine (52.9%) of the 17 subjects colonized at both sampling sites (nose/oropharynx) carried the same isolate in the nasal and oral mucosa, while eight (47.0%) carried different *S. aureus* strains in the nose and throat. Interestingly, two of the 17 subjects concomitantly colonized with *S. aureus* in the nose and throat had their strains (nasal and oropharyngeal) typed after digestion with different restriction enzymes. Isolates 691O and 747 N were typed with *SmaI*, while 691 N and 747O could only be typed with *ApaI*, confirming that they are different *S. aureus* strains. There was also one patient colonized with MRSA (735 N) in the nasal mucosa and with MSSA (735O) in the oropharyngeal mucosa, both typed with *ApaI*.

Analysis of typable isolates with *ApaI* revealed three major *clusters* (A, C, and D) and three minor *clusters* of 3 isolates (B, E, and F). In *cluster* A which contained 14 isolates with 85.6% similarity, two isolates were from the nasal and oropharyngeal mucosa of the same patient and the remaining from different subjects. The same was observed for *clusters* C and D. In *cluster* D, the isolates were grouped with a strain previously identified in another study as ST398 (strain 76 N). These findings are shown in Supplementary Material Figure S2.

### Molecular typing of *S. aureus* and MRSA by multilocus sequence typing (MLST)

Based on the *clusters* obtained by PFGE, nine *S. aureus* isolates (four MSSA and five MRSA) were selected for molecular typing by the MLST technique.

Typing of the MRSA isolates revealed a predominance of sequence type ST5 in three of the four isolates analyzed and one isolate with ST8. Regarding SCC*mec* type, isolates of the following lineages were obtained: ST5-IV (n = 2), ST5-I (n = 1), and ST8-IV (n = 1). In addition, the strain typed with *ApaI* by PFGE was characterized as ST398-IV (Fig. 2).

**Fig. 3:**
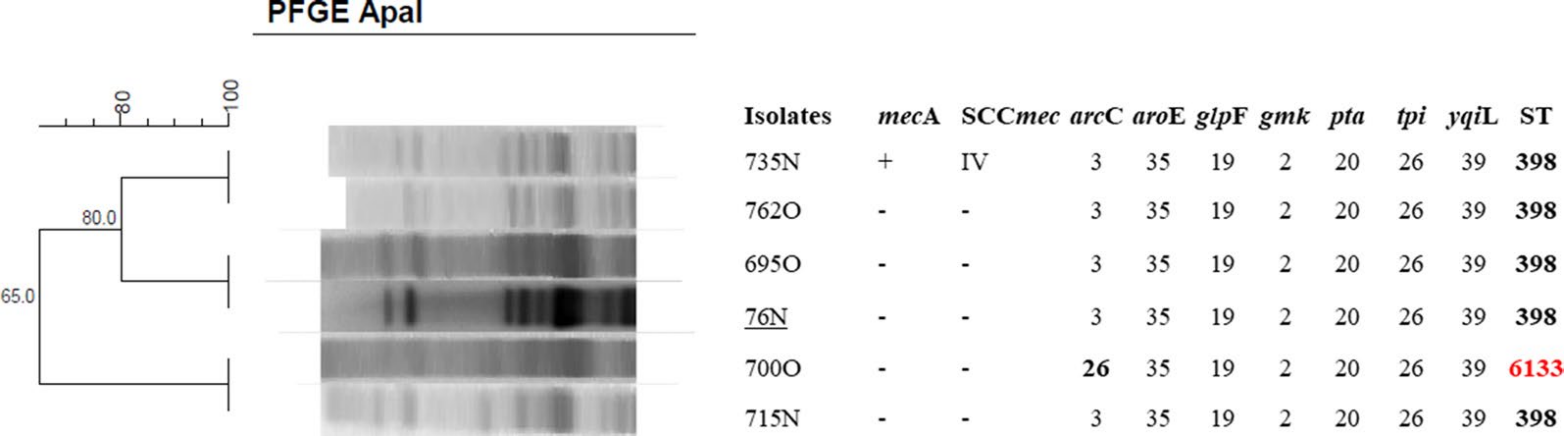
Dendrogram of the PFGE-*ApaI* profiles of MSSA isolates generated by Dice analysis/UPGMA (BioNumerics, Applied Maths) and sequence types obtained by MLST. Clustering of isolates digested with *ApaI* that were analyzed by MLST. All isolates except for 735 N were susceptible to methicillin (MSSA). N, nasal mucosa; O, oropharyngeal mucosa; *arc*C, carbamate kinase; *aro*E, shikimate dehydrogenase; *glp*F, glycerol kinase; *gmk*, guanylate kinase; *pta*, phosphate acetyltransferase; *tpi*, triosephosphate isomerase; *yqi*L, acetyl coenzyme A; ST, sequence type. Isolate 76 N was identified as ST398 in a previous study from our group. Isolate 700O was sent to the curator of the MLST database (https://pubmlst.org/) for identification of the new ST. This isolate was identified as ST 6133

Among the *clusters* of the MSSA isolates obtained by PFGE that could not be typed with *SmaI* and that were digested with *ApaI*, four lineages were selected for MLST. There was a predominance of ST398 (n = 3). However, one isolate exhibited divergence in the allele of the *arc*C gene and was sent to the curator of the MLST database (https://pubmlst.org/) for identification of the ST. This isolate was identified as ST 6133 (Fig. 3).

## Discussion

The prevalence of colonization with *S. aureus* and MRSA among insulin-dependent diabetic individuals was 30.4% and 4.8%, respectively. Similar data have been reported by Hart et al. [37] who analyzed 258 patients and found a prevalence of 39.1% for *S. aureus* and 3.1% for MRSA among diabetic individuals. In a population-based survey conducted in the same city, Pires et al. [38] found a similar prevalence among community-dwelling individuals, with an overall prevalence of *S. aureus* of 32.7%. However, the prevalence of MRSA was higher in our study (4.8% vs 0.9%). In a recent study, Lin et al. [2] found a lower prevalence of *S. aureus* and MRSA than that obtained in the present study (16.4% *S*. *aureus* and 2.8% MRSA). The authors suggested that the presence of different microorganisms in the microbiota of these individuals causes competition for the same site, which could explain the variation found in prevalence studies. None of the studies included oral mucosa as a potential site of colonization.

It should be noted that 10.9% (n = 34) of the subjects were colonized exclusively in the oral mucosa, six of them with MRSA. This finding reinforces the suggestion of Partida et al. [39] that colonization of the oral mucosa can compromise control measures of pathogen dissemination since the throat is not part of routine screening.

The MRSA isolates were identified by phenotypic methods (disc diffusion) and by PCR for detection of the *mec*A gene. We found four isolates carrying the *mec*A gene that did not exhibit phenotypic resistance to cefoxitin or oxacillin. Although *mec*A gene resistance is present in all cells of a population with intrinsic resistance, it may only be expressed by a small proportion of these cells, a fact that results in the so-called heteroresistance [40]. Isolates carrying the *mec*A gene but that are susceptible to oxacillin/cefoxitin have been reported worldwide and are called oxacillin-susceptible MRSA (OS-MRSA) [41–45]. According to Andrade-Figueiredo & Leal-Balbino [46], this phenomenon may be due to partial excision of SCC*mec* in multidrug-resistant MRSA isolates or chromosomal integration of the cassette chromosome, resulting in MSSA isolates that contain SCC*mec* segments.

High antimicrobial resistance of MRSA isolated from diabetic patients has been reported in different studies [14–16]. In our study, there were no multidrug-resistant isolates and none of the isolates was resistant to vancomycin, although one MRSA isolate had a MIC of 1.5 μg/mL, indicating a potential therapeutic risk [47–49]. Two other MSSA isolates had a vancomycin MIC of 1.5 μg/mL.

In the present study, the analysis of risk factors revealed an association only with lower-extremity ulcers, which is consistent with literature findings showing that the same isolate colonizing the nares was present in foot ulcers and wounds [50, 51].

Age was associated with a lower risk of *S. aureus* colonization, with a 2% decrease in the risk of colonization for each additional year of age. Similar data have been reported by Pereira-Franchi et al. [52]. It is believed that, with increasing age, individuals are exposed to factors (not analyzed in our study) that can prevent colonization with *S. aureus*, in addition to increased ecological competition with other microorganisms.

Lung disease was also a protective factor against the acquisition of *S. aureus*, a fact that might be related to colonization of the respiratory tract with other microorganisms that are competing with *S. aureus*. The nasopharyngeal microbiota changes over time; the level of bacterial colonization is higher during upper respiratory infection [53] and other species such as *Streptococcus pneumoniae* and *Haemophilus influenzae* may thus interfere with the capacity of *S. aureus* to persist in the nasal mucosa. Mueller et al. [54] also found a protective effect of age but, in contrast to our findings, lung disease was a risk factor for *S. aureus* colonization.

Multivariate analysis identified male gender as a risk factor for colonization with MRSA. Similar results were verified in a study of the prevalence of *S. aureus* and MRSA in bedridden individuals and residents of Long-Term Care Institutions for the Elderly (ILPIs) in the same city [55]. In this study developed by Silva [55], the male gender was associated with increased risk for the carrying of *S. aureus* and was also the only variable that showed to be a risk factor for carrying MRSA. Nillius et al. [56] reported that male ILPI residents had an almost double risk for carrying MRSA when compared to women, probably because they had more risk factors than they did.

With respect to clonality of the MRSA isolates, lineages belonging to the most widespread clonal complexes were identified, including CC5-ST5-IV (639O) and CC8-ST8-IV (72O) isolated from individuals with colonized oropharyngeal mucosa. This fact reinforces the importance of throat colonization, which could be a route of transmission within the population examined. Other studies involving individuals from the same city and region also found CC5-ST5-IV and CC8-ST8-IV, suggesting that these strains are prevalent in the region [57–62].

Studies suggest a high clonal diversity among *S. aureus* isolates, particularly among MSSA [63]. Among the isolates that could not be typed with *SmaI*, four were typed by MLST and were characterized as ST398. This fact was also observed by de Souza [61]. The ST398 clonal lineage has been associated with infection and colonization of humans and domestic animals, such as dogs, horses and pigs, in many countries around the world [8]. This lineage is called livestock-associated *S. aureus* and was described for the first time among both MSSA and MRSA on pig farms in France [64, 65]. Since then, ST398 has spread rapidly to other animals and has been increasingly related to infections not only in rural workers but also in people and animals without risk factors [31, 66]. Although susceptible to oxacillin, this *S. aureus* lineage is associated with severe infections, as reported by Bonesso et al. [67] in patients with ventilator-associated pneumonia in whom the infection was fatal in most cases.

Our finding demonstrated a predominance of SCC*mec* type IV among isolates, in agreement with the findings of other prevalence studies on non-diabetic individuals conducted in the State of São Paulo [57, 61]. However, SCC*mec* types I and II were also detected, which are commonly found circulating in health services. This fact has also been reported by Pereira-Franchi et al. [62] and Silveira et al. [68] who found a higher prevalence of isolates harboring SCC*mec* type II, which was attributed to a history of hospitalization.

It is worth mentioning that patients in hospital-community settings, such as bedridden or institutionalized older adults with chronic infections, have a higher prevalence of SCC*mec* types I and II [62, 68]; in addition, hospitalized patients frequently carry isolates that harbor SCC*mec* type III. These SCC*mec* are larger and carry plasmids and transposons with other resistance genes, often multidrug-resistant genes. On the other hand, community-dwelling patients are associated with SCC*mec* types IV and V, which are smaller and carry only the *mec*A gene of methicillin resistance [38].

One limitation of the present study is the small number of MRSA isolates (n = 15), which may result in a low statistical power of the analyses. In addition, the patients studied are not typically community-dwelling since they often need to seek health services (primary care) because of their diabetes.

The present study provides important data about the epidemiology of *S. aureus* and MRSA in a population of insulin-dependent diabetic individuals. The isolates analyzed had a low rate of resistance to the tested drugs, with only one isolate being resistant to sulfamethoxazole-trimethoprim; however, the prevalence of MRSA was higher than that found in a population-based study conducted in the same city on healthy individuals [38]. Within this context, screening for oral colonization is extremely important since some individuals were colonized only at this body site. In the population studied here, clones were detected among the MSSA and MRSA isolates and an important clonal lineage (ST398) was identified. These data suggest widespread dissemination of MRSA in the population of insulin-dependent diabetic patients studied, as well as the emergence of important *S. aureus* lineages in these individuals.

## Supplementary information

**Additional file 1.** They were included as tables of the characteristics of the individuals included and excluded from the study. In addition to the dendrograms of the PFGE-*Sma*I and PFGE-*Apa*I profiles of MSSA isolated from insulin-dependent diabetic individuals.

## Data Availability

The datasets used and analyzed during the current study are available from the corresponding author on reasonable request.
