# Supplementary material for: Molecular characterization of methicillin-resistant Staphylococcus aureus among insulin-dependent diabetic individuals in Brazil
Source: Ann Clin Microbiol Antimicrob. 2021 Feb 10;20:12. doi: 10.1186/s12941-020-00401-y (PMC7876813; doi:10.1186/s12941-020-00401-y)
Supplement: Supplementary file 1 — Additional file 1. They were included as tables of the characteristics of the individuals included and excluded from the study. In addition to the dendrograms of the PFGE-SmaI and PFGE-ApaI profiles of MSSA isolated from insulin-dependent diabetic individuals. [file 12941_2020_401_MOESM1_ESM.docx]

**Supplementary Material**

In this session, they were included as tables of the characteristics of the individuals included and excluded from the study. In addition to the dendrograms generated for the MSSA obtained.

**Appendix 1**

**Formula used for sample size calculation**

In Botucatu, 1,631 individuals with insulin-dependent diabetes are registered with the Municipal Health Department to receive a glucose meter, corresponding to the base population of our study. A representative sample was selected based on the following parameters:

- Total population: 1,631.
- Estimate of *S. aureus* carriage (p): 50% (value suggested for unknown proportion).
- Margin of error (d): 5%.
- Design effect (DEFF): 1.

Based on these parameters, application of the formula n = [DEFF*Np(1-p)]/[(d2/Z21-α/2*(N-1)+p*(1-p)] resulted in a suggested sample of 312 subjects.

**Table S1. Number of isolates according to the location of sample collection.**

| **Samples collected** | **n (%)** |
| --- | --- |
| At home | 204 (65.4) |
| At Basic Health Units* | 70 (22.4) |
| In the doctor’s office* | 23 (7.4) |
| At ABAD* | 15 (4.8) |
| Total | 312 (100) |
| * The individuals were contacted by telephone and invited to come to the location of sample collection.  ABAD: Botucatu Association for Diabetes Support | |

**Table S2. Number of excluded patients according to the exclusion criteria.**

| **Exclusion criterion** | **n (%)** |  |
| --- | --- | --- |
| Individuals not found based on personal data* | 388 (82.5) | |
| Individuals who did not use insulin | 70 (14.9) |  |
| Deaths | 10 (2.2) |  |
| Refusal of sample collection | 2 (0.4) |  |
| Total | 470 (100) |  |
| *The personal data of the insulin-dependent diabetic individuals were provided by the Municipal Health Department.. | |  |

**Table S3.** Antimicrobial susceptibility profile of MSSA and MRSA isolates.

| ***S. aureus* (112)** | ***mec*A gene** | **Oxacillin**  **R S** | **Cefoxitin**  **R S** | **Linezolid**  **R S** | **Q/D**  **R S** | **S/T**  **R S** | **Vancomycin MIC* (μg/mL)** |
| --- | --- | --- | --- | --- | --- | --- | --- |
| MSSA (97) | 0 | 0 97 | 0 97 | 0 97 | 0 97 | 1 96 | 0.19 – 1.5 |
| MRSA (15)  **Total (112)** | 15  15 | 7 8  7 105 | 11 4  11 101 | 0 15  0 112 | 0 15  0 112 | 0 15  1 111 | 0.38– 1.5  0.19 – 1.5 |

**Note.** Isolates identified as MSSA and MRSA based on the presence of the *mec*A gene. R= resistant; S= susceptible; Q/D=quinupristin/dalfopristin; S/T= sulfamethoxazole/trimethoprim; MIC = minimum inhibitory concentration. *Range of vancomycin susceptibility obtained for *mec*A-negative (MSSA) and *mec*A-positive (MRSA) isolates.


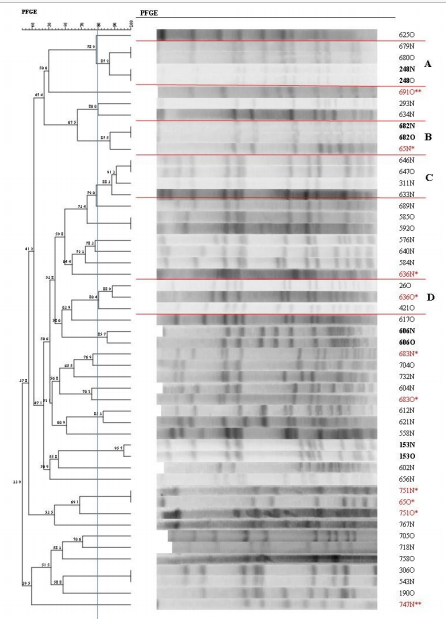


**Note.** Isolates in bold are from the same subject and have the same profile at both sampling sites (nasal/oropharyngeal). * Isolates obtained from the same subject but showing different profiles. ** The isolates identified as 691N and 747O and obtained from the same subjects as isolates 691O and 747N, respectively, were not typed with *Sma*I. N = nasal mucosa; O = oropharyngeal mucosa.

**Figure S1.** Dendrogram of the PFGE-*Sma*I profiles of MSSA isolated from insulin-dependent diabetic individuals generated by Dice analysis/UPGMA (BioNumerics, Applied Maths).


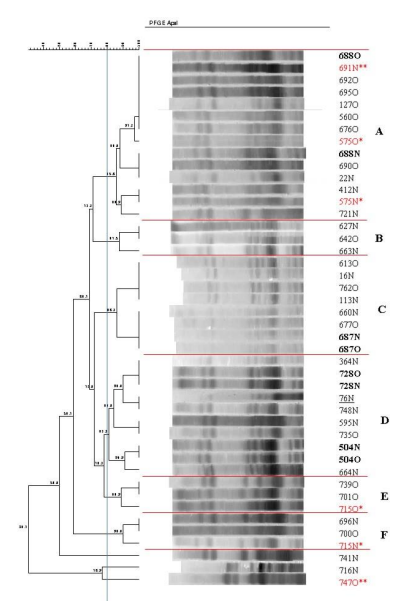


**Note.** Isolates in bold are from the same subject and have the same profile at both sampling sites (nasal/oropharyngeal)). * Isolates obtained from the same subject but showing different profiles. ** The isolates identified as 691N and 747O and obtained from the same subjects as isolates 691O and 747N, respectively, were typed with *Sma*I. Strain 76N previously isolated in another study and identified as *S. aureus* ST398 in a patient from the same city is underlined in the dendrogram. N = nasal mucosa; O = oropharyngeal mucosa.

**Figure S2.** Dendrogram of the PFGE-*Apa*I profiles of MSSA isolated from insulin-dependent diabetic individuals generated by Dice analysis/UPGMA (BioNumerics, Applied Maths).
